# Supplementary material for: Detection of cell‐free DNA fragmentation and copy number alterations in cerebrospinal fluid from glioma patients
Source: EMBO Mol Med. 2018 Nov 6;10(12):e9323. doi: 10.15252/emmm.201809323 (PMC6284385; doi:10.15252/emmm.201809323)
Supplement: Supplementary file 1 — Appendix [file EMMM-10-e9323-s001.pdf]

Appendix file

Detection of cell-free DNA fragmentation and copy number alterations in cerebrospinal fluid from glioma patients

Florent Mouliere1,2,3,*, Richard Mair1,2,4,*, Dineika Chandrananda1,2, Francesco Marass1,2,5,6, Christopher G. Smith1,2, Jing Su1,2, James Morris1,2, Colin Watts4, Kevin Brindle1,2,7,#, Nitzan Rosenfeld1,2,#.

Table of contents

Appendix Figure S1: SCNAs plots from the 13 CSF samples

Appendix Figure S2: Detailed SCNAs plot of the different tumor sub-parts and fluids (CSF, plasma, urine) available for patient GB1

Appendix Figure S3: Comparison of fragment length distributions for 13 glioma patient CSF samples.

Appendix Table S1: Summary table of the patients included in the study.

Appendix Figure S1 SCNAs plots from the 13 CSF samples with SCNAs detected (A), and without SCNAs detected (B).

A

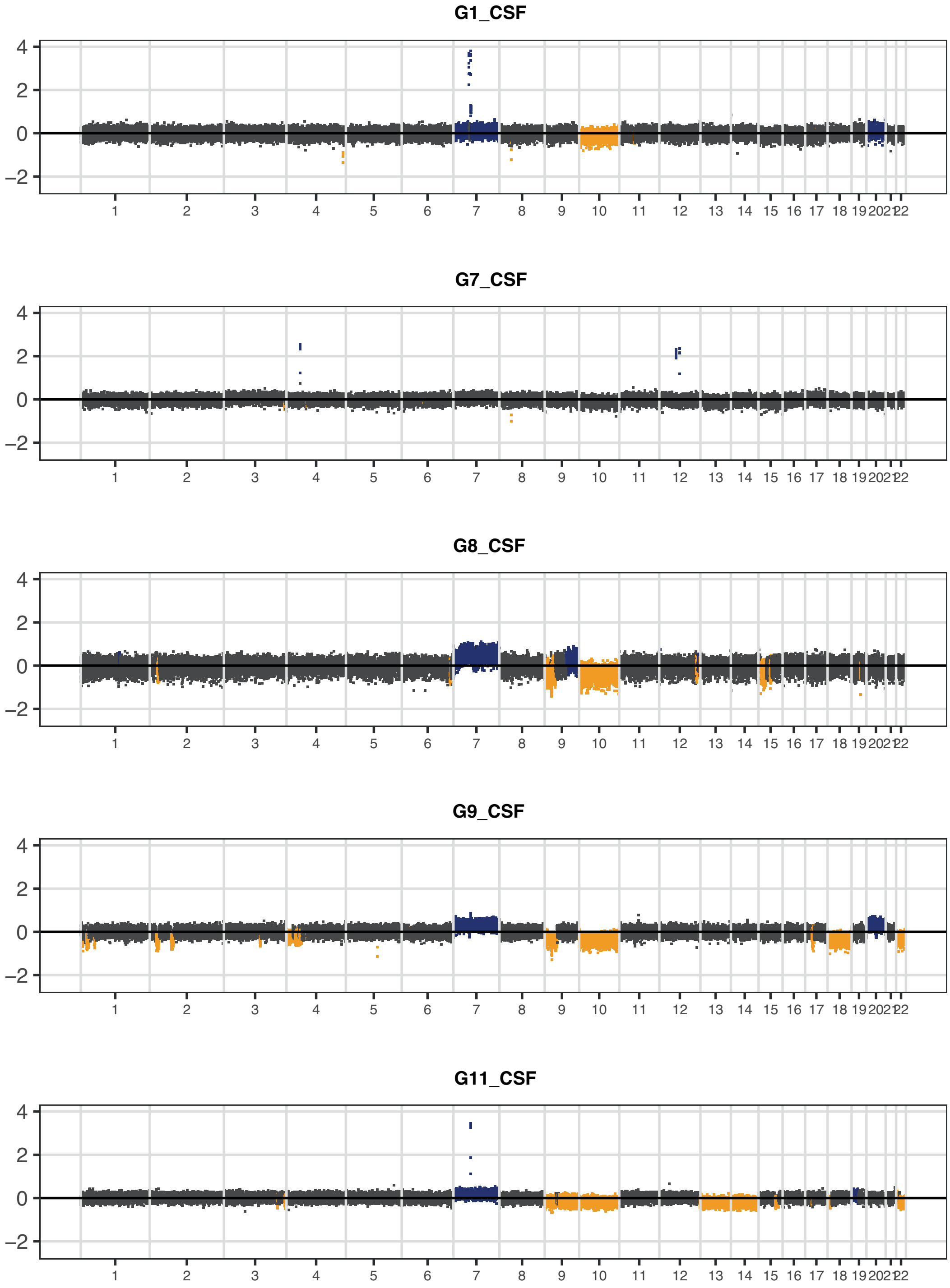

B

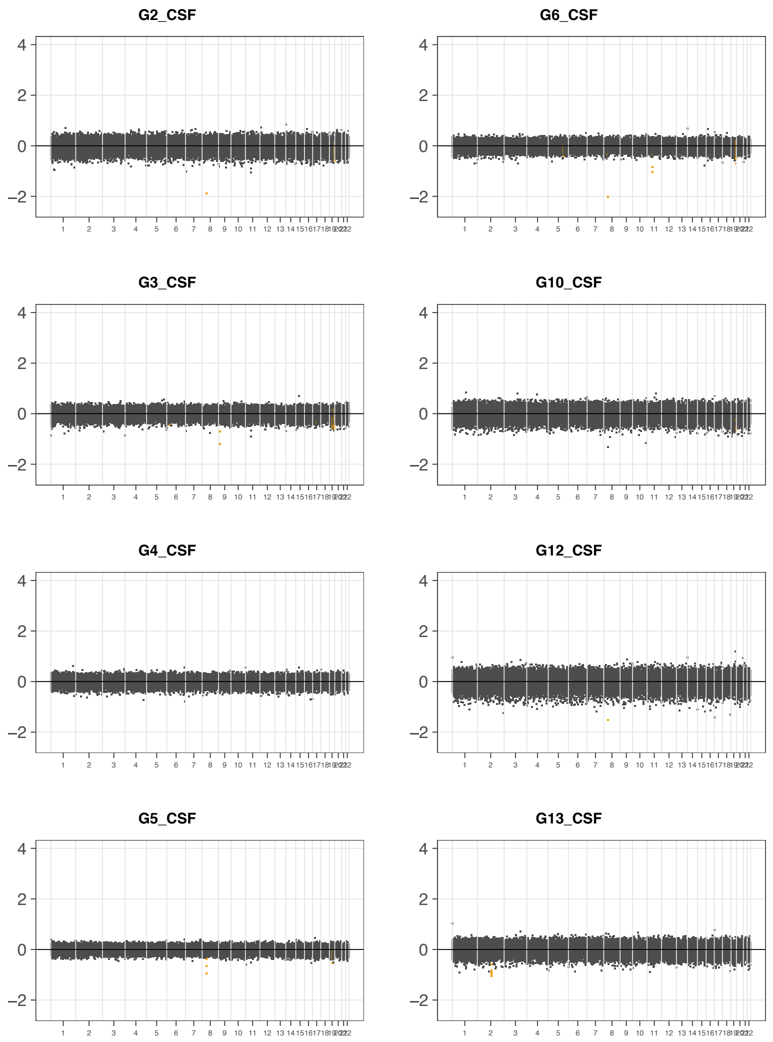

G2_CSF
G6_CSF
G3_CSF
G10_CSF
G4_CSF
G12_CSF
G5_CSF
G13_CSF
4
2
0
−2
1 2 3 4 5 6 7 8 9 10 11 12 13 14 15 16 17 18 19 20 21 22

Appendix Figure S2

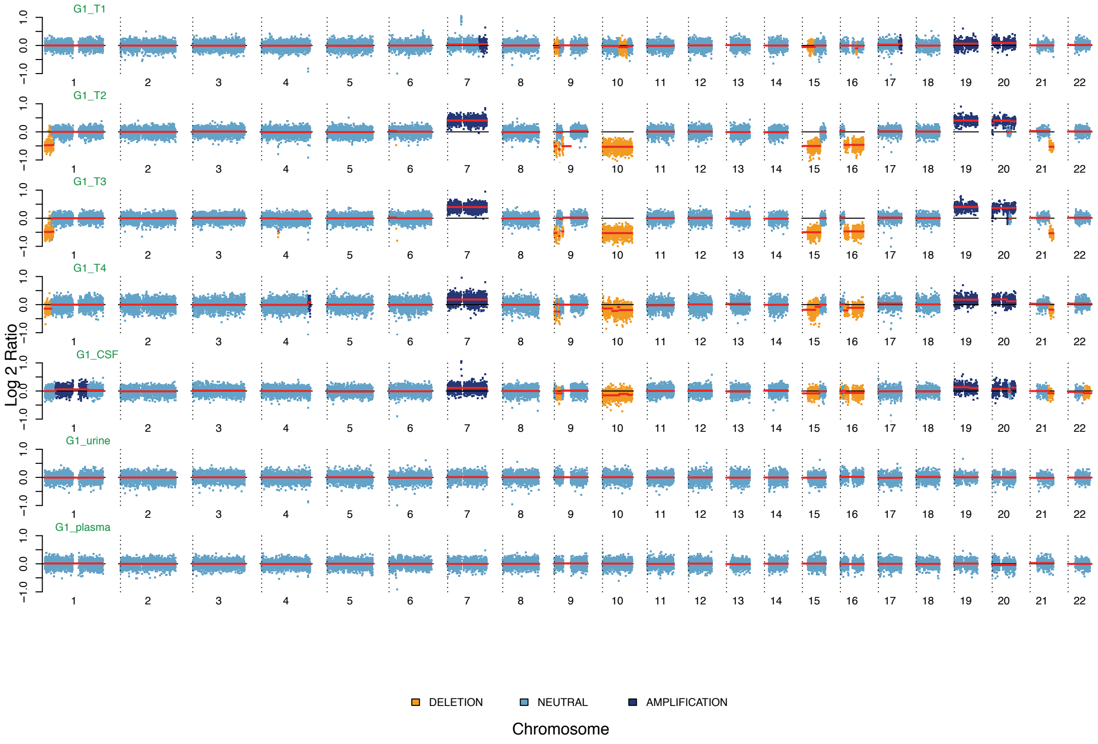

Appendix figure S2 Detailed SCNAs plot of the different tumor sub-parts and fluids (CSF, plasma, urine) available for patient GB1. Amplification are in dark blue, deletion in orange and copy number neutral regions in light blue.

Appendix figure S3 Comparison of fragment length distributions for 13 glioma patient CSF samples. The color of the size profile indicates whether SCNAs are detected (in blue) or non-detected (in red). Two patients have a solitary peak at ~145bp (G8 and G13) with one patient expressing a solitary peak at ~133bp (G1). Six patients display bimodal peaks at ~145 and ~167bp (G2, G6, G9, G5, G10 & G12). Four patients display peaks at ~167bp only (G11, G3, G4 & G7).

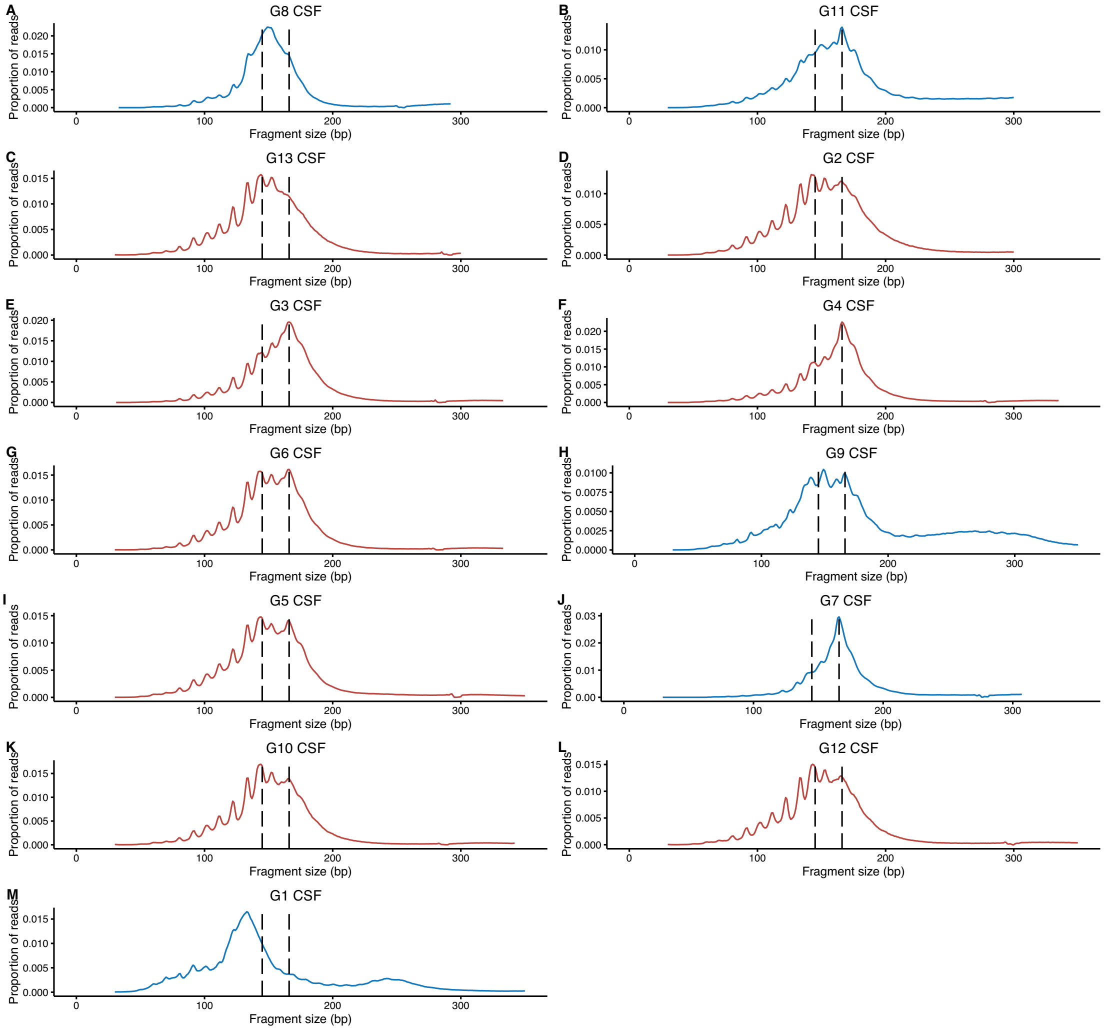

Appendix Table 1 Summary table of the patients included in the study.

patient	tumor type	tumor grade	tumor size (maximum dimension - mm)	cfDNA copies per mL	in contact with CSF	SCNA detected in CSF	ctDNA detected by fragmentation alone
G7	Glioblastoma	4	55	2446	Y	1	1
G9	Glioblastoma	4	44	1786	Y	1	1
G11	Glioblastoma	4	50	653	Y	1	1
G5	Glioblastoma	4	64	384	Y	0	0
G1	Glioblastoma	4	86	257	Y	1	1
G2	Glioblastoma	4	39	256	Y	0	0
G13	Glioblastoma	4	41	247	Y	1	0
G12	Anaplastic oligodendroglioma	3	27	234	Y	0	0
G10	Oligodendroglioma	2	30	132	Y	0	0
G8	Glioblastoma	4	33	102	N	1	1
G3	Diffuse astrocytoma	2	81	64	Y	0	0
G4	Glioblastoma	4	35	64	Y	0	0
G6	Glioblastoma	4	29	0	Y	0	0
